# High MERS-CoV seropositivity associated with camel herd profile, husbandry practices and household socio-demographic characteristics in Northern Kenya

**DOI:** 10.1017/S0950268820002939

**Published:** 2020-12-01

**Authors:** I. Ngere, P. Munyua, J. Harcourt, E. Hunsperger, N. Thornburg, M. Muturi, E. Osoro, J. Gachohi, B. Bodha, B. Okotu, J. Oyugi, W. Jaoko, A. Mwatondo, K. Njenga, M. A. Widdowson

**Affiliations:** 1 Washington State University Global Health Program, Nairobi, Kenya; 2 Division of Global Health Protection, US Centers for Disease Control and Prevention-Kenya, Nairobi, Kenya; 3 Division of Viral Diseases, National Center for Immunization and Respiratory Diseases, US Centers for Disease Control and Prevention, Atlanta, GA, USA; 4 Kenya Ministry of Agriculture Livestock and Fisheries, Zoonotic Disease Unit, Nairobi, Kenya; 5 Department of Veterinary and Livestock, County Government of Marsabit, Marsabit, Kenya; 6 Department of Veterinary Services, County Government of Marsabit, Marsabit, Kenya; 7 Department of Medical Microbiology, University of Nairobi, Nairobi, Kenya; 8 Kenya Ministry of Health, Zoonotic Disease Unit, Nairobi, Kenya; 9 Institute of Tropical Medicine, Antwerp, Belgium; 10 School of Public Health, Jomo Kenyatta University of Agriculture and Technology, Nairobi, Kenya

**Keywords:** Kenya, Middle East respiratory syndrome coronavirus, nomadic communities, seroprevalence, zoonoses

## Abstract

Despite high exposure to Middle East respiratory syndrome coronavirus (MERS-CoV), the predictors for seropositivity in the context of husbandry practices for camels in Eastern Africa are not well understood. We conducted a cross-sectional survey to describe the camel herd profile and determine the factors associated with MERS-CoV seropositivity in Northern Kenya. We enrolled 29 camel-owning households and administered questionnaires to collect herd and household data. Serum samples collected from 493 randomly selected camels were tested for anti-MERS-CoV antibodies using a microneutralisation assay, and regression analysis used to correlate herd and household characteristics with camel seropositivity. Households reared camels (median = 23 camels and IQR 16–56), and at least one other livestock species in two distinct herds; a home herd kept near homesteads, and a range/fora herd that resided far from the homestead. The overall MERS-CoV IgG seropositivity was 76.3%, with no statistically significant difference between home and fora herds. Significant predictors for seropositivity (*P* ⩽ 0.05) included camels 6–10 years old (aOR 2.3, 95% CI 1.0–5.2), herds with ⩾25 camels (aOR 2.0, 95% CI 1.2–3.4) and camels from *Gabra* community (aOR 2.3, 95% CI 1.2–4.2). These results suggest high levels of virus transmission among camels, with potential for human infection.

## Introduction

Middle East respiratory syndrome coronavirus (MERS-CoV) is a zoonotic respiratory infection that is endemic in dromedary camels (*Camelus dromedarius*) and causes asymptomatic or mild-to-severe illness in the human population. Evidence supporting camels as the primary reservoir includes isolation of the virus, the high prevalence of MERS-CoV antibodies in camel sera from many countries in the Middle East, Africa and Asia, and the ability to be experimentally infected with the virus [1–5]. Since 2012, when the first human case of MERS-CoV was detected in Saudi Arabia, there have been 2494 confirmed human cases and 858 deaths reported from 27 countries, including the Middle East, Southeast Asia, Europe, North America and North Africa [6, 7]. Interestingly, despite the high prevalence of MERS-CoV in camels in sub-Saharan Africa and human contact with camels and camel products, no transmission events from camels to humans have been identified in the region [8, 9].

Human-to-human transmission of MERS-CoV can occur with close contact, with over half of the confirmed human cases worldwide occurring in healthcare settings [10]. However, human-to-human transmissions, associated with casual contact, or household settings are rare [11, 12]. Thus, camel-to-human transmission is generally considered an important pathway as supported by the geographical linking of genetically similar camel and human MERS-CoV isolates [3]. While the exact mechanism of primary MERS-CoV infection to humans from camels remains poorly understood, viral genomic studies suggest much higher rates of camel-to-human than human-to-human virus migration [13]. The MERS-CoV infection in humans causes severe respiratory illness and death in up to 35% of cases, while infection in camels is usually sub-clinical with occasional cases of mild rhinitis [14]. The two other coronaviruses that cause clinical illness in humans, including severe acute respiratory syndrome coronavirus 1 and 2 (SARS-CoV-1 and SARS-CoV-2) which are also zoonotic, have caused some of the most devastating pandemics and epidemics in human history [15, 16].

In the camel population, MERS-CoV appears to spread efficiently, with over 70% seroprevalence reported among camels in Kenya and other parts of the world [17–19]. A longitudinal study in Egypt reported the virus spread to >90% of a herd within 2 months, followed by a rapid decline in antibodies following the end of the outbreak to suggest that multiple re-infections of herds can occur within a short period [18]. Younger camels are particularly susceptible to MERS-CoV infection when compared with older ones, shed more virus and could drive infections in camel herds [20]. The Horn of Africa, including Kenya, Sudan, Ethiopia and Somalia, is home to over 70% of the global dromedary camels population, and camel movement from the region to the Arabian Peninsula exceeds 300 000 animals every year through trade [21, 22]. Studies showed that camels imported from the Horn of Africa had higher MERS-CoV prevalence than Arabian peninsula camels, suggesting that importations played a role in the introduction and maintenance of the virus in the Arabian peninsula [18, 23]. Because of this, the spread of MERS-CoV has threatened camel trade, which is a major source of livelihood for the nomadic pastoralists in the Horn of Africa [24]. Currently, there are no drugs or vaccines for the management of MERS-CoV infections in humans or camels, although several molecules and treatments are currently being trialled for the human disease [25].

A limited number of cross-sectional surveys have shown mixed results of MERS-CoV seropositivity [2, 19, 26–28]; however, the determinants of these variations in seropositivity among camel-keeping communities in Kenya have not been fully elucidated [18, 27]. Here, we describe the herd profile and determinants of MERS-CoV seropositivity among camels owned by the nomadic pastoralist communities living in Northern Kenya.

## Materials and methods

### Study site and setting

**Fig. 1.**
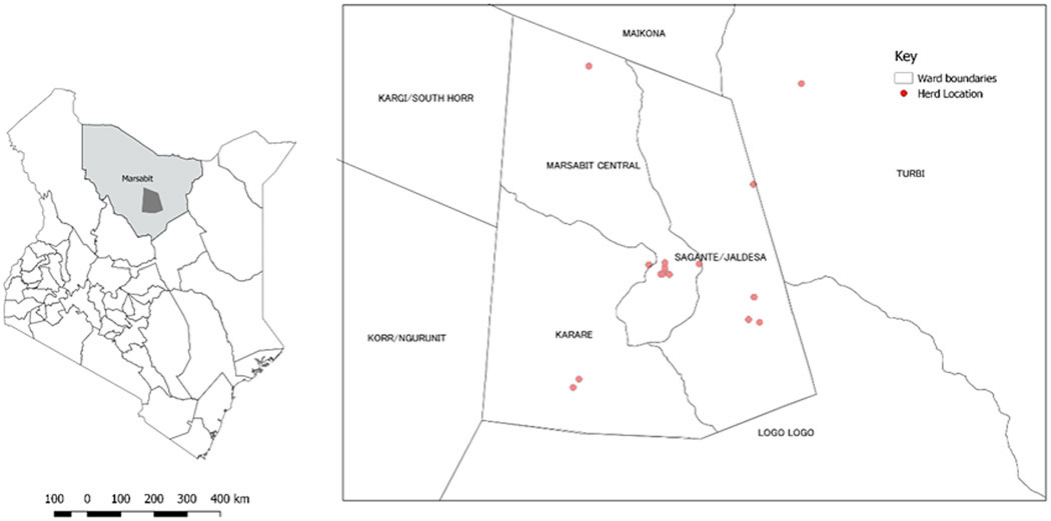
Location of the study area in Marsabit County with the spatial distribution of sampled herds (red dots) within Saku sub-county. This map was drawn on QGIS Version 2.18.15 using mapping resources from the International Livestock Research Institute (ILRI) [29].

Figure 1 shows the location of Marsabit County in Northern Kenya with camel sampling sites (red dots) in homesteads, grazing fields and watering points. Marsabit County is the largest county in Kenya, located in the sparsely populated arid regions of the country. The county is mainly inhabited by nomadic pastoralists from the Gabra, Borana and Rendille communities living in *Manyattas* (traditional villages) in geographically defined regions. The study was conducted in Marsabit central, in areas inhabited by the three communities. Most households in these communities keep livestock including cattle, goats, sheep, donkeys and chicken, and over 60% of the households own camels [30].

### Study design, sample size and camel selection

We carried out a cross-sectional study in July–August 2018, immediately following the long rainy season. We estimated a minimum sample size of camels (*n*) using the formula *n* = (1.96)^2^*p*(1 − *p*)/*d*^2^, with a margin error (*d*) of 0.05 at 95% confidence level [31]. We assumed a seroprevalence (*p*) of 70% based on previous studies [27] and a design effect of 1.5 to account for cluster sampling. Through participatory epidemiology approaches such as mapping of human settlement and livestock movement patterns with camel farmers in the study area and informant interviews with community leaders, we identified and enlisted 29 camel-owning households living in nine settlements/villages for inclusion in the study (Supplementary Table S1). Herd owners or their representatives were provided with information about the study and then interviewed upon consenting. Owing to the nomadic lifestyle of the households, sampling of camels in each settlement was concluded in one day. On the day of sampling, the number of camels in each of the nine settlements was estimated through participatory consultations with camel owners and herders. A sampling interval (**I**) was computed by dividing the estimated population of camels for each settlement by the daily target of 54 camels. A systematic random sampling approach was adopted whereby the first camel was identified by drawing a random number between one and **I**, then successive **I**^th^ camels were identified and sampled in each herd until the household proportion was met. The number of camels sampled in each household in the settlement was proportional to the size of the household's camel herd. Since some households owned more than one camel herd, only the herds found near the settlement/village at the time of visit were sampled.

### Sample collection

After adequately restraining the camels, 10 ml of blood was collected from the jugular vein into a plain vacutainer tube (BD Vacutainer®, product 366430). Samples were labelled and transported in frozen ice packs at +4 °C from the field to the laboratory at Marsabit veterinary department for processing. Clotted samples were centrifuged at 2795 g for 6 min and extracted sera aliquoted into two 1.8 ml barcoded cryovial tubes (Thermofisher Scientific^TM^ product 375418) followed by storage at −20 °C in the field before being transferred to −80 °C freezer in Kenya Medical Research Institute/Centers for Global Health Research (KEMRI-CGRH) laboratories in Nairobi for long-term storage. One aliquot was shipped to the CDC Respiratory Virus Immunology Laboratory in Atlanta, USA, for testing of MERS-CoV IgG antibodies using a microneutralisation assay (MNT).

### MERS-CoV microneutralisation assays

Prior to testing, samples were *γ* irradiated by exposure to 5 × 10^6^ rad of ^60^Co, to inactivate potential pathogenic contaminants, and then heat-inactivated at 56 °C for 30 min. MERS-CoV MNT was performed following biosafety level-3 precautions using a clinical isolate of MERS-CoV (Hu/Jordan-N3/2012) provided by the Jordan Ministry of Health and Naval Medical Research Unit 3 (Cairo, Egypt) [32, 33]. Vero cells (ATCC CCL-81) were prepared at 2 × 10^5^ cells/ml in DMEM (Life Technologies, product 11965118) + 10% foetal bovine serum (Hyclone) and incubated at 37 °C and 5% CO_2_ until a confluency of 85–95% was achieved. In a 96-well flat-bottom plate, sera were diluted to a final concentration of 1:20 in serum-free DMEM including 1× penicillin-streptomycin (Gibco Life Technologies, product 15140122) to a final volume of 50 μl. The virus was diluted to a final working dilution of 200 TCID_50_/ml in serum-free cell culture media, and 50 μl added to each well. Following 30 min incubation at 37 °C and 5% CO_2_, Vero cells were added to each well at a final concentration of 2 × 10^4^ cells/well. After 5 days further incubation, cell culture plates were fixed, stained and scored. In a level-3 biosafety cabinet, media was aspirated from wells, and 150 μl crystal violet fixative (0.15% crystal violet, 2.5% ethanol, 11% formaldehyde, 50% PBS, 0.01 M pH 7.4) added to each well. Plates were incubated in a biosafety cabinet for 20 min at 20–22 °C. Fixative was aspirated, plates washed and scored. Each serum specimen was tested in triplicate and was considered positive for MERS-CoV antibodies if at least two of three replicate wells were protected against virus infection (specimen had to remain completely purple following staining with crystal violet). A 1:20 dilution was used as a lower limit of detection for positive results. Positive and negative control sera from previously tested camels were used in each MNT as described previously [33, 34]. At 1:20, all three wells of negative controls remained negative, and positive controls remained positive.

### Data collection and statistical analysis

Trained research assistants used a structured questionnaire preloaded into Android^®^ tablets running the RedCap^®^ data collection platform to collect data on household socio-demographic information, geographic location, livestock herd structure, camel production and camel herd management information. Data analysis was carried out using R statistical software, version 3.3.3 (R Core Team, 2013). Mean or median values were computed for continuous variables and reported where appropriate after an independent assessment of normality using visual inspection and Shapiro–Wilk's test. Proportions and their 95% confidence intervals (CIs) were computed and reported for categorical variables. Analysis of the determinants of seropositivity was carried out at individual camel and herd levels to determine the association between MERS-CoV seropositivity and individual camel factors, herd structure and husbandry practices, and household socio-demographic factors in univariate and multivariate analysis. The *χ*^2^ test was performed for categorical data and odds ratios (ORs) with corresponding 95% CIs calculated while adjusting for intra-herd clustering and number of camels sampled in each herd (herd unique ID included as a random effect). A *P*-value of <0.05 was considered significant. Significant predictors with *P*-value ⩽0.1 in the univariate analysis were selected and included in the multivariate model in a stepwise process through forward and backward selection while adjusting for herd clustering (herd unique ID as a random effect). We first constructed a full model with all variables selected from the univariate analyses and proceeded to drop those with *P* > 0.05 based on Wald's *χ*^2^ test. We then sequentially re-introduced the dropped variables (smallest *P*-values first) and retained them in the model only if the *P*-value remained ⩽0.05. The 95% CIs were computed for the adjusted OR with a *P*-value of <0.05 considered significant in the final model.

## Results

### Socio-demographic characteristics of herd owners

We enrolled a total of 29 herd owners, each representing a household, of which 44.8% (*n* = 13) were from the Borana community and 27.6% (*n* = 8) each from Gabra and Rendille communities. All respondents were male, median age of 44 years (IQR 35.52) and 75.9% (*n* = 22) had no formal education. The median size of households was 10 persons (IQR 6–11) and 82.7% (*n* = 24) reported an average monthly income of US$100 or more (Table 1).

**Table 1.** Socio-demographic characteristics of respondents and households in Marsabit (*n* = 29)

Socio-demographic variables	*N* (%)
Median age of household head, years (IQR)	44 (35–52)
Ethnic community of origin of the household head	
Borana	13 (44.8)
Gabra	8 (27.6)
Rendille	8 (27.6)
Sex (male herd owner)	29 (100.0)
Highest level of formal education	
No formal education	22 (75.9)
Primary and above	7 (24.1)
Religion	
Christian	15 (51.7)
Muslim	11 (37.9)
Traditional	3 (10.4)
Occupation	
Unemployed	9 (31.0)
Employed	20 (69.0)
Median family size (IQR)	10 (6–11)
Household average monthly income	
<US$100	5 (17.3)
US$100–499	17 (58.6)
US$500*+*	7 (24.1)

IQR, interquartile range; US$, US dollar.

### Camel herd profile, herd dynamics and herding practices in Marsabit County

All 29 households reared camels and at least one other livestock species; 25 (86.2%) owned goats, 21 (72.4%) owned cattle and sheep, and 13 (44.8%) owned donkeys (Table 2). Goats constituted the largest herds (*n* = 2983) with a median of 45 goats (IQR 18–135), followed by camels (*n* = 1343, median = 23 and IQR 16–56), sheep (*n* = 1541, median = 12 and IQR 5–100), cattle (*n* = 554, median = 10 and IQR 7–27) and donkeys (*n* = 67, median 5 and IQR 4–7). Overall, 75.8% (22/29) of all households owned at least three livestock species. Camel ownership was primarily through family inheritance, and the average lifespan of a camel in a household was 17.5 years (standard deviation = 15.0). The larger Somali camel breed was commonly reared among households and comprised 44.5% (598/1343) of all the camels owned by enrolled households, whereas the smaller Gabra and Rendille breeds comprised 32.2% (433/1343) of the camels. Camel breed preference was based on hardiness (14.5%), high milk production (29.0%), high resale value (18.8%), faster growth and high meat production among others (10.1%) (Supplementary Table S2). A total of 408 (30.4%) camels were newly acquired and introduced into the herds in the year preceding the study, representing an average of 14 newly sourced camels per herd (Table 2). Livestock were grazed in communal lands and watered in shared watering points with clearly defined boundaries that geographically delineated the Gabra, Borana and Rendille grazing lands. The watering points were shared by members of the same community on a rotational basis throughout the year depending on the season and forage cover (Supplementary Table S3).

**Table 2.** Livestock herd structure, composition and herding practices in Marsabit

Variable	*N* (%)
Number of households by livestock ownership, *n* (%)	*n* *=* 29
Camel	29 (100.0)
Goats	25 (86.2)
Cattle	21 (72.4)
Sheep	21 (72.4)
Donkeys	13 (44.8)
Median size of livestock herds, IQR (Q1–Q3)	
Goats (*n* = 2983)	45 (18–135)
Camels (*n* = 1343)	23 (16–56)
Sheep (*n* = 1541)	12 (5–100)
Cattle (*n* = 554)	10 (7–27)
Donkeys (*n* = 67)	5 (4–7)
Number of livestock species per household, *n* (%)^a^	*n* *=* 29
Five species	3 (10.3)
Four species	8 (27.6)
Three species	11 (37.9)
Two species	7 (24.1)
Camel herd by breed, *n* (%)	*n* *=* 1343
*Somali*	598 (44.5)
*Gabra/Rendille*	433 (32.2)
Breed not known	312 (23.3)
New camel introductions per herd (camels/herd) *n* = 408^b^	14
Median number of camels by age group, IQR (Q1–Q3)	
6 months and below	5 (1–8)
7–12 months	4 (2–6)
1 year and above	17 (10–47)
Median number of camels by herd separation, IQR (Q1–Q3)	
Home herd^c^	4 (0–37)
Fora herd^d^	20 (10–63)
Average camel lifespan in years (s.d.)	17.5 (15.0)

IQR, interquartile range; s.d., standard deviation.

a *Number of livestock species per household*: five livestock species were considered, camels, cattle, goats, sheep and donkeys. Only one household had poultry.

b *New camel introductions per herd*- computed for the 1-year period preceding the study by dividing the number of camels newly acquired by the number of herds. Camels were acquired through purchase, gifting, inheritance and as dowry payment.

c ‘*Home herd*’ refers to camels ordinarily reared at home/settlement mainly for sustenance of the households.

d ‘*Fora herd*’ refers to the herd that roams away from home/settlement in search of pastures.

Camel-owning households reared their camels either in a home herd (*n* = 8), a range (or fora) herd (*n* = 13) or in both home and range herds (*n* = 8). The home herd was comprised of lactating female camels, dams in late pregnancy and calves of both sexes <1 year of age (median herd size = 4, IQR 0–37). This herd was maintained closer to the homestead with access to nearby water sources and pastures and attended to by young boys or older members of the household. The home herd was the milking herd, providing milk for domestic consumption and sale. The fora herds, which comprised of male calves >1 year of age and non-pregnant or non-lactating dams, were larger (median size = 20, IQR 10–63 and *P* < 0.01), and migrated up to 150 km from home in search of water and pastures. Older boys and hired herders looked after the fora herd. The home and fora herds were reunited at the homestead during the rainy season when pastures were plenty, and during a biennial traditional community celebration referred to as the *Sorrio* ceremony. The *Sorrio* ceremony, observed for 3 weeks by all the three communities of Marsabit, follows the lunar calendar and involves slaughtering and sharing of camel meat, smearing of camel blood as a mark of communal blessing, and free mixing of home and fora herds of the entire village.

### MERS-CoV seropositivity and associated factors

**Fig. 2.**
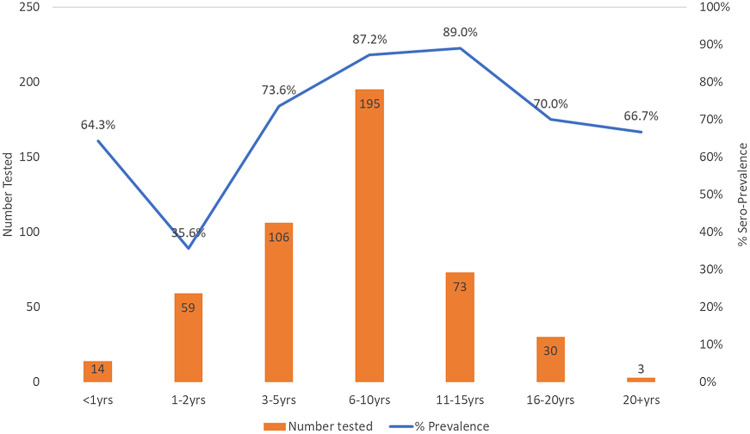
Distribution of MERS-CoV seropositivity in camels by age categories, 2018. Dataset includes serum samples from 493 randomly selected camels from among 29 herds in Marsabit Central, Kenya. Samples were tested for anti-MERS-CoV antibodies using a microneutralisation assay and the % seropositivity (blue line) and the number of camels tested (orange bars) were plotted against camel age categories as shown in the chart.

A total of 493 camel serum samples from 29 herds were collected and tested. The mean age of sampled camels was 7.4 years (s.d. = 4.8). Of the 493 samples, 376 (76.3%) samples tested positive for anti-MERS-CoV neutralizing antibodies. All the 29 herds reported seropositive animals and camel herd-level IgG seropositivity ranged from 26.7% to 100% among the sampled camels. Stratified by age category, MERS-CoV seropositivity dropped from 64.3% in camels aged below 1 year to 35.6% for camels aged 1–2 years. Thereafter, seropositivity increased progressively, reaching 89.0% for camels aged 11–15 years. Beyond 16 years of age, seropositivity decreases to 70.0% then to 66.7% for camels aged 20 years or more (Fig. 2).

The distribution of seropositivity by camel age, sex, and household and herd characteristics is as shown in Table 3. Although the mean age of sampled camels was 7.4 years (s.d. = 4.8), seropositive camels were older (mean age 7.9 years, s.d. = 4.5 and *P*-value <0.001), when compared to seronegative camels (mean age = 5.9, s.d. = 5.7). On univariate analysis, seropositivity significantly varied by age categories, sex, respondents ethnic background, average household monthly income and camel herd sizes. Camel age was found to be a significant predictor of camel-level seropositivity (*P* < 0.05) with more camels aged 6–10 years old (OR 3.0, 95% CI 1.3–6.9) and 11–15 years old (OR 3.5, 95% CI 1.2–10.0) being exposed than younger camels aged 1–2 years (OR 0.2, 95% CI 0.1–0.6) when compared to camels aged 16 or more years. Similarly, female camels (when compared to males), camels from *Gabra* and *Rendille* households (compared to *Borana* households), camels from large herds (⩾25 camels) and camels from households with low monthly income (below US$100) had significantly increased risk of being seropositive (Table 3).

**Table 3.** Univariate analysis of individual camel, herd and household socio-demographic factors associated with MERS-CoV seropositivity among camels in Marsabit (*n* = 493)

Variable	Seronegative *n* = 117(%)	Seropositive *n* = 376(%)	Total *N* = 493(%)	Crude OR (95% CI)^a^	*P*-value^b^
Camel age group (years)					
<1	5 (4.2)	9 (2.4)	14 (2.9)	0.8 (0.2–2.9)	0.742
1–2	38 (32.5)	21 (5.6)	59 (12.2)	0.2 (0.1–0.6)	**0.002**
3–5	28 (23.9)	78 (20.7)	106 (21.5)	1.2 (0.5–2.9)	0.661
6–10	25 (21.4)	170 (45.2)	195 (39.6)	3.0 (1.3–6.9)	**0.009**
11–15	8 (6.8)	65 (17.3)	73 (15.1)	3.5 (1.2–10.0)	**0.014**
16+	10 (0.9)	23 (5.6)	33 (6.7)	1 (Ref^c^)	
Sex of camel (*n* = 487)					
Female	84 (71.8)	320 (85.1)	404 (83.0)	2.4 (1.4–4.1)	**0.002**
Male	32 (27.4)	51 (13.6)	83 (17.0)	1 (Ref)	
Highest education level of camel owner					
None	103 (88.0)	324 (86.2)	427 (89.9)	1 (Ref)	
Primary and above	14 (12.0)	52 (13.8)	66 (10.1)	1.2 (0.5–2.8)	0.710
Ethnic background of the herd owner					
Borana	61 (52.1)	101 (26.9)	162 (32.9)	1 (Ref)	
Gabra	35 (29.9)	196 (52.1)	231 (46.9)	3.4 (1.6–7.0)	**0.001**
Rendille	21 (17.9)	79 (21.0)	100 (20.3)	2.3 (1.3–4.1)	**0.007**
Average household monthly income					
US$0–99	31 (26.5)	72 (19.1)	103 (20.9)	0.4 (0.2–0.8)	**0.007**
US$100–499	69 (59.0)	204 (54.3)	273 (55.4)	1.5 (0.7–3.2)	0.301
US$500+	11 (9.4)	35 (9.3)	46 (9.3)	1 (Ref)	
Frequency of relocation in the last 1 month					
Do not move	28 (23.9)	118 (31.4)	146 (29.6)	1 (Ref)	
Move from place to place	89 (76.1)	258 (68.6)	347 (70.3)	0.7 (0.3–1.5)	0.358
Herd separation					
Home herd	49 (41.9)	146 (38.8)	195 (39.5)	1 (Ref)	
Fora herd	68 (58.1)	230 (61.2)	298 (60.5)	1.1 (0.6–2.2)	0.707
Camel herd sizes					
<25 Camels per herd	61 (52.1)	95 (25.3)	156 (31.6)	1 (Ref)	
25 and above camels per herd	56 (47.9)	281 (74.7)	337 (68.4)	3.2 (1.8–5.8)	**<0.001**
Duration of camel keeping (years)					
<10	22 (18.8)	79 (21.0)	101 (25.3)	1 (Ref)	
10–20	44 (37.6)	104 (27.7)	148 (37.1)	0.7 (0.3–1.6)	0.342
>20	32 (27.4)	118 (31.4)	150 (37.6)	1.1 (0.5–2.1)	0.881

US$, US dollar; 95% CI, lower and upper limits for 95% confidence intervals.

a Categorical variables evaluated using *χ*^2^ test and crude odds ratios and corresponding *P*-values shown. Results of Fisher's exact test (FET) reported for few observations.

b *p* < 0.05 was considered significant and is shown in bold type.

c Reference category. Normative categories were selected as the reference groups for variables such as camel sex, highest education level, frequency of nomadism and herd separation while the largest or smallest category was selected for variables such as camel age, household income level and duration of camel keeping. The first alphabetic category was used to select reference category for ‘ethnic background’ while for camel age, the reference group was selected from the oldest age category since younger age has been shown as a significant determinant of seropositivity in past studies.

Seven factors that had *P*-values ⩽0.1 were included in the multivariate model, while adjusting for herd clustering. The model identified camels aged between 6 and 10 years, camels from Gabra households and camels from larger herds of ⩾25 camels as important predictors of camel-level MERS-CoV seropositivity (Table 4).

**Table 4.** Multivariate analysis of individual camel, herd and household socio-demographic factors associated with MERS-CoV seropositivity among camels in Marsabit

Variable	Crude OR (95% CI)	*P*-value^a^	Adjusted OR (95% CI)	*P*-value
Age of camel 6–10 years	2.6 (1.2–6.0)	**0.022**	2.3 (1.0–5.2)	**0.05**
Age of camel 11–15 years	2.2 (0.8–5.7)	0.110	1.7 (0.7–4.5)	0.22
Female camel	2.4 (1.4–4.1)	**0.002**	1.8 (1.0–3.5)	0.06
Camel owner is from *Gabra* community	3.4 (1.6–7.0)	**0.001**	2.3 (1.2–4.2)	**<0.01**
Camel owner is from *Rendille* community	2.3 (1.3–4.1)	**0.007**	1.5 (0.8–2.7)	0.22
Monthly income 0–99	0.4 (0.2–0.8)	**0.007**	0.1 (0.1–3.5)	0.29
Large camel herd (⩾25 camels)	3.2 (1.8–5.8)	**<0.001**	2.0 (1.2–3.4)	**0.01**

a *p* < 0.05 was considered significant and is shown in bold type

## Discussion

In this survey, we describe the household demographic and socio-economic factors, the livestock and camel herd profile and also highlight the determinants of individual and herd-level MERS-CoV seropositivity among camels in Northern Kenya. Overall, all herds sampled had seropositive camels, and animal-level seropositivity was associated with increasing age of camel (up to 10 years), large herd size (⩾25 camels) and ethnic community.

The herd-level and individual camel MERS-CoV seropositivity in the present study were comparable to the findings of previous studies in the same locality which used ELISA-based assays to detect MERS-CoV IgG antibodies in 74–90% of samples from Marsabit [2, 19, 27]. In a study of 85 camel herds from Northern Kenya by Munyua *et al*., the herd-level seropositivity was also 100% [19]. In a study of archived camel sera collected in Kenya between 1992 and 2013, the authors found higher exposure of camels to MERS-CoV that was associated with highly nomadic camels, while in a separate study of serum samples from nine camel herds in central Kenya, the authors found a much lower seroprevalence [2, 35].

Although we did not assess MERS-CoV exposure among human subjects in contact with the camels, the finding of high seroprevalence among camels would imply high circulation of the virus in this environment and the potential for camel-to-human transmission, as has been shown in studies that have isolated MERS-CoV virus in this regions [27, 28]. However, the findings from previous studies in this region suggest minimal zoonotic transmission of the local MERS-CoV strain [19]. Still, the high seroprevalence in the area could point to the existence of unique drivers of transmission and maintenance of the virus among camels in this environment.

The herd profile described here is diverse, comprising of large herds of mixed livestock species (average of 95 animals per household) with goats and camels forming the majority of animals in the herd. Households reared as many as five livestock species, including >75% of that owned three to five different species. This species diversification is deliberate, perhaps as an insurance against economic losses arising from recurrent droughts and devastating livestock disease outbreaks [36, 37]. Although different livestock species were reared together with the highly seropositive camels, we did not test sera from other livestock species for MERS-CoV IgG antibodies since they have been shown not to be susceptible to MERS-CoV infection in other studies [38–40].

Almost 75% of the camels in the herds were adults (>2 years old), most reared as fora herds that resided as far as 150 km away from the homestead. The fora herds travelled up to 40 km daily in search of pastures and water and, in the process, mixed with other herds. These fora herds return to the original homestead during the rainy seasons when the pastures are plenty, and during communal celebrations. This rotational use of land is primarily designed to ensure effective use of pastures, help with pest and disease control, and to prevent inter-ethnic conflicts [41]. This planned pattern of camel movement over vast pasturelands, coupled with the introduction of up to 30% new camels annually, means a high rate of camel transition, and interaction between herds that may promote and sustain MERS-CoV infection and transmission within these herds. Studies have shown that MERS-CoV spreads between camels following contact with respiratory droplets or aerosolised virus from the faeces of infected camels [3, 4, 42, 43].

We found more than threefold higher seroprevalence among older camels and those belonging to larger herd sizes (*P* < 0.001), characteristics more commonly in fora than home herds. Other studies have documented that MERS-CoV spreads more rapidly and efficiently in large herds, sometimes affecting the entire herd within a short period [44]. Larger camel herds are likely to offer a mix of infected camels that are shedding the virus and non-immune animals, creating an environment for a sustained transmission and maintenance of the virus [18, 45]. An important question that is not fully addressed is how long immunity to MERS-CoV lasts in camels, even though few studies suggest that the immunity may be short-lived [18]. We did not find any significant differences in the seroprevalence of anti-MERS-CoV antibodies between home and range camel herds (77.2% *vs.* 74.1%, *P* = 0.7). Despite being reared separately, these herds mixed frequently during communal ceremonies. Studies have demonstrated that differences in livestock production and management systems may affect the spread of infections [46, 47].

In agreement with other studies, we documented a large proportion of calves 0–12 months of age with high neutralizing antibodies, suggesting the persistence of maternal antibodies [14, 18, 44]. The high seroprevalence observed in camels owned by the Gabra (>3-fold) and Rendille (>2-fold) communities may be explained by differences in camel herd structures and husbandry practices. We found female camels having higher seropositivity than males; however, this association reduced in magnitude in the multivariate model. It is likely that once maternally derived antibodies wane later in the first year of life, calves become infected repeatedly, thereby sustaining MERS-CoV infection in the herd and maybe infecting pregnant and lactating camels [18, 48, 49].

Our study had a few limitations. The small number of herds (*N* = 29) in the survey limited our ability to draw further inferences on herd and community characteristics. Second, aspects of this study including specific risk factors for MERS-CoV transmission such as communal ceremonies (*Sorrio*) and human contact with camels could be better studied through mixed-methods studies. Our study used an MNT to determine the presence of anti-MERS-CoV antibodies in camel sera. An MNT was preferred for two reasons. First, the assay allowed for evaluation of the presence of functional antibodies in camel sera that recognise MERS-CoV and prevent infection *in vitro*. Second, the MNT effectively reduced non-specific binding and high positive signals in camels that have other cross-reacting antibodies (including antibodies to bovine coronaviruses), a challenge that may not have been easily overcome by most ELISA assays available to us [33]. However, it is possible that the neutralisation assay missed certain samples that produced non-neutralizing antibodies and those newly infected before the production of detectable levels of antibodies. A camel's seropositivity to anti-MERS-CoV antibodies indicates exposure and does not imply that the animal had active infections at the time of sampling, which may distort some of the associations observed.

## Conclusion

Our study highlights the determinants of high MERS-CoV seropositivity in camels in the context of different camel husbandry practices (small *vs.* large herds, home *vs.* fora herds, young *vs.* older camels) in Northern Kenya, the knowledge that is needed in addressing the occupational risk of MERS-CoV among camel handlers. The MERS-CoV exposure profile depicted in our study suggests high transmission of the virus, primarily within the currently known reservoir camel population, underlining the need for camel vaccine to address potential human exposure. In the absence of alternative control strategies, it would be important to determine the duration of immunity in natural camel infections, as this could have a direct impact on the frequency and success of vaccination programmes in camels.

## Data Availability

All data generated or analysed during this study are included in this published article and its supplemental files.

## Supplementary material

For supplementary material accompanying this paper visit https://doi.org/10.1017/S0950268820002939. click here to view supplementary material
