# Supplementary material for: High MERS-CoV seropositivity associated with camel herd profile, husbandry practices and household socio-demographic characteristics in Northern Kenya
Source: Epidemiol Infect. 2020 Dec 1;148:e292. doi: 10.1017/S0950268820002939 (PMC7737118; doi:10.1017/S0950268820002939)
Supplement: Supplementary file 1 [file S0950268820002939sup001.zip › S0950268820002939sup001.docx]

# Epidemiology and Infection

# Camel herd profile and risk factors for MERS-CoV seropositivity in Northern Kenya

### Authors

*I. Ngere, P. Munyua, J. Harcourt , E. Hunsperger, N. Thornburg, M. Muturi , E. Osoro, J. Gachohi , B. Bodha , B. Okotu , J. Oyugi , W. Jaoko , A. Mwatondo , K. Njenga , MA. Widdowson*

# Supplementary table 3

**Supplementary Table 3: Camel herding practices among communities in Marsabit County**

| **Variable** | **Total**  N(%) |  |
| --- | --- | --- |
| Average distance to herding fields |  |  |
| *<5Km (Less than an hour walk)* | 9 (31.0) |  |
| *5-10Km (1-2hour walk)* | 8 (27.6) |  |
| *>10Km (More than 2 hours walk)* | 12 (41.4) |  |
| Who does the herding |  |  |
| *Farm worker/Employee* | 18 (47.4) |  |
| *Son* | 12 (31.5) |  |
| *Other** | 8 (21.1) |  |
| Herding Role |  |  |
| *Specific person* | 23 (79.3) |  |
| *Any person* | 6 (20.70 |  |
| Frequency of nomadism in the last 1 month |  |  |
| *Don’t move* | 8 (27.6) |  |
| *Moved once* | 13 (44.8) |  |
| *Moved more than once* | 8 (27.6) |  |
| Reasons for Nomadism |  |  |
| *To look for pasture* | 12 (29.3) |  |
| *To look for water* | 11 (26.80 |  |
| *To escape pests/diseases* | 8 (19.5) |  |
| *Other** | 10 (24.4) |  |
| Who makes decision to move with camels |  |  |
| *Herd owner/Self* | 17 (81.0) |  |
| *Other** | 4 (19.0) |  |
| * Other person involved in herding included relatives (1) and male household head (7)  * Other reasons for nomadism included to escape insecurity (1), poor weather conditions (4), to be close to market (2) and as a way of life (3) | | |
