# Supplementary material for: High MERS-CoV seropositivity associated with camel herd profile, husbandry practices and household socio-demographic characteristics in Northern Kenya
Source: Epidemiol Infect. 2020 Dec 1;148:e292. doi: 10.1017/S0950268820002939 (PMC7737118; doi:10.1017/S0950268820002939)
Supplement: Supplementary file 1 [file S0950268820002939sup001.zip › S0950268820002939sup002.docx]

# Epidemiology and Infection

# Camel herd profile and risk factors for MERS-CoV seropositivity in Northern Kenya

### Authors

*I. Ngere, P. Munyua, J. Harcourt , E. Hunsperger, N. Thornburg, M. Muturi , E. Osoro, J. Gachohi , B. Bodha , B. Okotu , J. Oyugi , W. Jaoko , A. Mwatondo , K. Njenga , MA. Widdowson*

# Supplementary table 2

**Supplementary Table 2: Camel herd management practices in Marsabit county**

| **Variable** | **Total**  N(%) |  |
| --- | --- | --- |
| Median duration of camel keeping in years (IQR) | 15.0 (18.0) |  |
| Reasons for keeping camels |  |  |
| *For Meat* | 11 (17.5) |  |
| *For milk* | 28 (44.4) |  |
| *For sale and business* | 19 (30.2) |  |
| *Other reason* | 5 (7.9) |  |
| Preferred Camel breed |  |  |
| *Somali* | 22 (75.9) |  |
| *Gabra/Rendile* | 7 (24.1) |  |
| Reason for camel breed preference |  |  |
| *Camel breed hardy* | 10 (14.5) |  |
| *Breed has higher milk production* | 20 (29.0) |  |
| *Breed has faster growth* | 7 (10.1) |  |
| *Profitable during sale* | 13 (18.8) |  |
| *Other** | 19 (27.5) |  |
| Who does the milking |  |  |
| *Male household head* | 13 (30.2) |  |
| *Farm worker/herder* | 15 (34.9) |  |
| *Son* | 11 (25.6) |  |
| *Other** | 4 (9.3) |  |
| Milking role |  |  |
| *Specific person* | 12 (41.4) |  |
| *Any person* | 17 (58.6) |  |
| Uses of Camel Milk |  |  |
| *Domestic consumption* | 10 (34.5) |  |
| *Sale* | 13 (44.8) |  |
| *Both (Sale & Domestic consumption)* | 6 (20.7) |  |
|  |  |  |
| * Other reasons included prestige/social status symbol, family tradition, for sport, drought resistant, providing for transport and easy management  * Other reasons for camel preference include only breed available (2), great load bearing ability (1), and family reasons (1)  * Other person included female spouse (2) and daughter (1)  *Other uses of camel milk included | | |
