# Supplementary material for: High MERS-CoV seropositivity associated with camel herd profile, husbandry practices and household socio-demographic characteristics in Northern Kenya
Source: Epidemiol Infect. 2020 Dec 1;148:e292. doi: 10.1017/S0950268820002939 (PMC7737118; doi:10.1017/S0950268820002939)
Supplement: Supplementary file 1 [file S0950268820002939sup001.zip › S0950268820002939sup003.docx]

# Epidemiology and Infection

# Camel herd profile and risk factors for MERS-CoV seropositivity in Northern Kenya

### Authors

*I. Ngere, P. Munyua, J. Harcourt , E. Hunsperger, N. Thornburg, M. Muturi , E. Osoro, J. Gachohi , B. Bodha , B. Okotu , J. Oyugi , W. Jaoko , A. Mwatondo , K. Njenga , MA. Widdowson*

# Supplementary table 1

### Supplementary Table 1: Details of herds that were enrolled and sampled in baseline survey

| **Sampling Location/Village** | **Number of herds** | **Estimated number of camels^a^** | **Calculated Sampling Interval^b^** | **No. of Camels sampled** |  |
| --- | --- | --- | --- | --- | --- |
| Hulahula | 6 | 224 | 4 | 40 |  |
| Thogogich On Bonsa | 2 | 63 | 1 | 23 |  |
| Simpir Galan | 2 | 171 | 3 | 65 |  |
| Gof Rable & Rable Finchani | 3 | 173 | 3 | 60 |  |
| Kubi On Renthila | 2 | 183 | 3 | 57 |  |
| Qachacha | 3 | 101 | 1 | 80 |  |
| Karare | 4 | 142 | 2 | 61 |  |
| Mude | 5 | 295 | 2 | 95 |  |
| Ilman Ngufu | 2 | 41 | 1 | 15 |  |
| **Totals** | **29** | **1393** |  | **496** |  |
| **^a^**- Total number of camels as verified from participatory consultation with the herd owners  **^b^**- sampling interval, estimated by dividing the estimated herd size by the daily target of 54 | | | | | |
