# Supplementary material for: High MERS-CoV seropositivity associated with camel herd profile, husbandry practices and household socio-demographic characteristics in Northern Kenya
Source: Epidemiol Infect. 2020 Dec 1;148:e292. doi: 10.1017/S0950268820002939 (PMC7737118; doi:10.1017/S0950268820002939)
Supplement: Supplementary file 1 [file S0950268820002939sup001.zip › S0950268820002939sup005.pdf]

Camel Herd Health Survey Questionnaire

Record ID	____________________
Interviewer ID:	◯ MC10 (Mutembei)<br>◯ MC11 (Denge)<br>◯ MC12 (Minayo)<br>◯ MC13 (Akoko)<br>◯ MC14 (Omollo)<br>◯ MC15 (Ngere)
Date of Interview	____________________

PART 1 - HOUSEHOLD IDENTIFICATION.

101. Name of County:	◯ Marsabit<br>◯ Other county
102. Name of Sub-county	◯ Marsabit Central (Saku)<br>◯ Marsabit North (Chalbi)<br>◯ Marsabit South (Laisamis)<br>◯ Loiyangalani<br>◯ Moyale<br>◯ Sololo
103. Name of ward	◯ Sagante/Jaldesa<br>◯ Karare<br>◯ Marsabit Central
104. Name of village	____________________

105. Household number

◯ M01
◯ M02
◯ M03
◯ M04
◯ M05
◯ M06
◯ M07
◯ M08
◯ M09
◯ M10
◯ M11
◯ M12
◯ M13
◯ M14
◯ M15
◯ M16
◯ M17
◯ M18
◯ M19
◯ M20
◯ M21
◯ M22
◯ M23
◯ M24
◯ M25
◯ M26
◯ M27
◯ M28
◯ M29
◯ M30
◯ M31
◯ M32
◯ M33
◯ M34
◯ M35
◯ M36
◯ M37
◯ M38
◯ M39
◯ M40
◯ M41
◯ M42
◯ M43
◯ M44
◯ M45
◯ M46
◯ M47
◯ M48
◯ M49
◯ M50
◯ M51
◯ M52
◯ M53
◯ M54
◯ M55
◯ M56
◯ M57
◯ M58
◯ M59
◯ M60
◯ M61
◯ M62
◯ M63
◯ M64
◯ M65
◯ M66
◯ M67
◯ M68
◯ M69

◯ M70
◯ M71
◯ M72
◯ M73
◯ M74
◯ M75
◯ M76
◯ M77
◯ M78
◯ M79
◯ M80
◯ M81
◯ M82
◯ M83
◯ M84
◯ M85
◯ M86
◯ M87
◯ M88
◯ M89
◯ M90
◯ M91
◯ M92
◯ M93
◯ M94
◯ M95
◯ M96
◯ M97
◯ M98
◯ M99

---

106. GPS coordinates (Latitude)

____

GPS coordinates (Longitude)

____

PART 2 - RESPONDENT'S BACKGROUND

201. Name of respondent:

____

---

202. What is the sex of the respondent?

◯ Male
◯ Female

---

203. Position in the household

◯ Household Head
◯ Spouse
◯ Farm worker
◯ Son/Daughter
◯ Other (Specify)

Please specify other position

____

---

204. Age of respondent

____

---

205. What is the highest level of education/school completed?

◯ Primary
◯ Secondary
◯ Tertiary
◯ No formal education

206. What is your religion?	◯ Christian<br>◯ Muslim<br>◯ Traditional<br>◯ No religion<br>◯ Other (specify)
Please specify other religion	__________________________________
207. Community of origin	◯ Borana<br>◯ Gabra<br>◯ Rendille<br>◯ Samburu<br>◯ Somali<br>◯ Turkana<br>◯ Other (Specify)
Please specify other community of origin	__________________________________

PART 3 - SOCIO-ECONOMIC STATUS

301. How many people live in this household?	__________________________________
302. How many people are under 15 years old in this household?	__________________________________
303. What is the main occupation of HH head? (tick where applicable)	◯ Formal employment<br>◯ Informal employment<br>◯ Self-employed formal<br>◯ Self-employed informal<br>◯ Unemployed<br>◯ Others (Please specify)
Specify the other occupation	__________________________________
304. What is the household's average monthly income?	◯ Ksh. 0 - 9,999<br>◯ Ksh. 10,000 - 29,000<br>◯ Ksh. 30,000 - 49,000<br>◯ Ksh. 50,000 - 99,999<br>◯ >Ksh. 100,000<br>◯ Refuse to answer<br>◯ Don't Know

PART 4: INFORMATION ON LIVESTOCK

401. How many camel(s) do you own?	__________________________________<br>(Give total number eg. 15)
402. Do you have camels in any of these age groups?	☐ 0-6 months<br>☐ 7- 12months<br>☐ 13+ months
0-6 months camels in HOME HERD?	__________________________________

0-6 months camels in OTHER HERD (Fora)? ______________________

7-12 months camels in HOME HERD? ______________________

7-12 months camels in OTHER HERD (Fora)? ______________________

13+ months camels in HOME HERD? ______________________

13+ months male camels in OTHER HERD (Fora)? ______________________

403. Apart from camels, do You have other livestock in this farm/herd?
- ◯ Yes
- ◯ No

404. If Yes, indicate the which of the following? (Indicate the number below)
- ☐ Cattle
- ☐ Sheep
- ☐ Goats
- ☐ Donkeys
- ☐ Poultry
- ☐ Other (specify)

Please indicate the number of cattle 0-12 months ______________________

Please indicate the number of cattle 13+ months ______________________

Please indicate the number of goats 0-12 months ______________________

Please indicate the number of goats 13+ months ______________________

Please indicate the number of sheep 0-12 months ______________________

Please indicate the number of sheep 13+ months ______________________

Please indicate the number of donkeys 0-12 months ______________________

Please indicate the number of donkeys 13+ months ______________________

Please indicate the number of poultry ______________________

Please specify other animals owned ______________________

Please indicate the number of other animals	__________________
405.Do you prefer camels over other livestock yes/no	◯ Yes<br>◯ No
If yes why?	☐ More drought resistant<br>☐ More profitable<br>☐ Easier to manage<br>☐ Habit<br>☐ Other (Specify)
Specify	__________________

PART 5: CAMEL REPRODUCTION QUESTIONS

501. At what age do your camels start breeding?	
501a. Male (Age in years when they start mounting females/rutting?)	__________________
501b. Females (Age in years at 1st calving)	__________________
502. At what age do the camels stop breeding?	
502a. Females (age in years whenthey stop calving)	__________________
503. What is the number of breeding camels?	
503a. Male	__________________
503b. Female	__________________
504. Which month is the breeding season for your herd?	☐ Bon Hagaya/Nabhay gaban/Short dry spell(January-March)<br>☐ Gan/Guh/Long rain(April-May)<br>☐ Bon/Nabhay Kider/Long dry spell(June -September<br>☐ Hagay/Yer/Short rain(October-December)
505a. What is the average number of years between one calving and the next?	__________________
505b. How many camels have calved in the last one year?	__________________
505c. At what age (in months)do calves start grazing/browsing?	__________________<br>(Enter age in Months eg 18)

505d. At what age (In months) do calves separate from the mother (stop suckling)? (Joining the fora)

____
(Give range in Months eg 7-8)

506. What mainly happens to the calves after separation from the mother?

506a. Males (after weaning)

- ◯ Retained in the herd
- ◯ Sold
- ◯ Slaughtered
- ◯ Other

Specify

____

506b. Females (after weaning)

- ◯ Retained in the herd
- ◯ Sold
- ◯ Slaughtered
- ◯ Other

Specify

____

507. Select 4 mature females that the herder/farmer is most familiar with (include the lead female camel) and ask the following questions for each camel

How many females have you selected?

- ◯ 1
- ◯ 2
- ◯ 3
- ◯ 4

Female Camel #1

Camel Name

____

Age in years

____
(Enter number only rg if 4 years, enter 4)

Reproduction status

- ◯ Pregnant
- ◯ Dry
- ◯ Lactating

Has this camel ever given birth before?

- ◯ Yes
- ◯ No

Date of last calving?

____

Sex of last calf

- ◯ Male
- ◯ Female

Common Calving Month(s)	☐ Bon Hagaya/Nabhay gaban/Short dry spell(January-March)<br>☐ Gan/Guh/Long rain(April-May)<br>☐ Bon/Nabhay Kider/Long dry spell(June -September<br>☐ Hagay/Yer/Short rain(October-December)<br>(Check All that Apply)
Last Calf Weaning age (in Months)	______________________<br>(Just the number eg. 24)
Lifetime calves	______________________<br>(Just the number eg. 2)
Dead calves	______________________
Lifetime abortions	______________________<br>(Just the number eg. 2)

Female Camel #2

Camel Name	______________________
Age	______________________<br>(Enter number only rg if 4 years, enter 4)
Reproduction status	◯ Pregnant<br>◯ Dry<br>◯ Lactating
Has this camel ever given birth before?	◯ Yes<br>◯ No
Date of last calving?	______________________
Sex of last calf	◯ Male<br>◯ Female
Common Calving Month(s)	☐ Bon Hagaya/Nabhay gaban/Short dry spell(January-March)<br>☐ Gan/Guh/Long rain(April-May)<br>☐ Bon/Nabhay Kider/Long dry spell(June -September<br>☐ Hagay/Yer/Short rain(October-December)<br>(Check All that Apply)
Last Calf Weaning age (in Months)	______________________<br>(Just the number eg. 24)

Lifetime calves

____
(Just the number eg. 2)

Dead calves

____

Lifetime abortions

____
(Just the number eg. 2)

Female Camel #3

Camel Name

____

Age

____
(Enter number only rg if 4 years, enter 4)

Reproduction status

○ Pregnant
○ Dry
○ Lactating

Has this camel ever given birth before?

○ Yes
○ No

Date of last calving?

____

Sex of last calf

○ Male
○ Female

Common Calving Month(s)

☐ Bon Hagaya/Nabhay gaban/Short dry spell(January-March)
☐ Gan/Guh/Long rain(April-May)
☐ Bon/Nabhay Kider/Long dry spell(June -September
☐ Hagay/Yer/Short rain(October-December)
(Check All that Apply)

Last Calf Weaning age (in Months)

____
(Just the number eg. 24)

Lifetime calves

____
(Just the number eg. 2)

Dead calves

____

Lifetime abortions

____
(Just the number eg. 2)

Female Camel #4

Camel Name

____

Age

____
(Enter number only rg if 4 years, enter 4)

Reproduction status

- ◯ Pregnant
- ◯ Dry
- ◯ Lactating

Has this camel ever given birth before?

- ◯ Yes
- ◯ No

Date of last calving?

____

Sex of last calf

- ◯ Male
- ◯ Female

Common calving month(s)

- ☐ Bon Hagaya/Nabhay gaban/Short dry spell(January-March)
- ☐ Gan/Guh/Long rain(April-May)
- ☐ Bon/Nabhay Kider/Long dry spell(June -September
- ☐ Hagay/Yer/Short rain(October-December)

(Check All that Apply)

Last Calf Weaning age (in Months)

____
(Just the number eg. 24)

Lifetime calves

____
(Just the number eg. 2)

Dead calves

____

Lifetime abortions

____
(Just the number eg. 2)

PART 6: CAMEL PRODUCTION QUESTIONS

601. Where did you get your camels from?

- ☐ Inherited
- ☐ Bought
- ☐ Came with family member
- ☐ Dowry (paid as bride price)
- ☐ Found
- ☐ Gifted to you
- ☐ Other (Specify)

Specify other place you got your camels

____

If inherited, how many?	______ (Just the number eg. 2)
If bought, how many?	______ (Just the number eg. 2)
If came with family member, how many?	______ (Just the number eg. 2)
If received from dowry/bride price, how many?	______ (Just the number eg. 2)
If found, how many?	______ (Just the number eg. 2)
If gifted to you, how many?	______ (Just the number eg. 2)
How many did you acquire through other sources?	______ (Just the number eg. 2)
602. Where did your camels originate from?	☐ In the village<br>☐ In the same sub-County<br>☐ In another County<br>☐ In another country<br>☐ Other
Other camel origin	______
603. For how long have you kept camels? (years)	______ (Enter number only eg. 3)
604. What are two primary reasons for keeping camels	☐ For Meat<br>☐ For milk<br>☐ For sale and business<br>☐ Prestige/social status<br>☐ Family tradition<br>☐ Part time activity<br>☐ For sport<br>☐ Other (specify)
Other reason for keeping camels	______
605. How do you dispose your camels? Tick all that apply	☐ Sell<br>☐ Slaughter<br>☐ Give as gifts<br>☐ Bride price<br>☐ Other (Specify)

Other method of disposing camels	__________________________
For selling, How many camels in the last year?	__________________________
For slaughtering, How many camels in the last year?	__________________________
For gifts, How many camels in the last year?	__________________________
For bride price, How many camels in the last year?	__________________________
For "other" disposal methods, How many camels in the last year?	__________________________
606. How many breeds of camel do you have (enter the numbers below)?	☐ Somali<br>☐ Turkana<br>☐ Gabra/Rendile<br>☐ Other
Number of Somali Breed	__________________________
Number of Turkana Breed	__________________________
Number of Gabra/Rendile Breed	__________________________
Other Breed (Specify here and write the total number below)	__________________________
Number for other camel species	__________________________
607. Of these camel breeds, which one do you prefer?	○ Somali<br>○ Turkana<br>○ Rendile/Gabra<br>○ None
Why do you prefer the above breeds/camel(s)?	☐ It is hardy<br>☐ Higher milk production<br>☐ Fast growth<br>☐ Mild temperament<br>☐ Great load bearer<br>☐ The only breed available<br>☐ Family reasons<br>☐ Other reason (Specify)
Please state other reason for preference	__________________________
608a. Did you slaughter any camel in the past one year?	○ Yes<br>○ No

608b. If yes, how many? ________________________________

608c. Please list the age of the first four camels you slaughtered below.

Camel 1 age slaughtered ________________________________

Camel 2 age slaughtered ________________________________

Camel 3 age slaughtered ________________________________

Camel 4 age slaughtered ________________________________

609. Whose role is it to milk the camels in your farm?
- ☐ Household Head
- ☐ Spouse
- ☐ Farm worker/Herder
- ☐ Son/Daughter
- ☐ Other (Specify

Specify other persons milking camels ________________________________

610. Whose role is it to milk?
- ◯ Specific person
- ◯ Any person

611. What is the main use of the milk from camels?
- ◯ Domestic Consumption
- ◯ For sale
- ◯ Other (Specify)

Other milk use? ________________________________

PART 7: CAMEL HEALTH QUESTIONS

701. Have you ever sought veterinary services for your herd?
- ◯ Yes
- ◯ No

702. If yes, how many times in a year do you usually seek veterinary services for your camel herd?
- ◯ Once
- ◯ Twice
- ◯ Thrice
- ◯ If more than 3 (specify)

Please specify if more than 3 times ________________________________

703. Who provides the veterinary services to your camel herd?
- ☐ Private
- ☐ Gov.
- ☐ Community Animal Health Workers
- ☐ Other (specify)

Specify other veterinary service provider ________________________________

704. How much do you pay on average per visit from the veterinarian / animal health technician?	◯ < Ksh. 500<br>◯ Ksh. 501 - Ksh. 5000<br>◯ >Ksh. 5001
705. How much did you spend in treatment of camels in the last 3 months?	◯ < Ksh. 500<br>◯ Ksh. 501 - Ksh. 5000<br>◯ >Ksh. 5001
706. If no, why have you never sought veterinary services for your camel(s)?	☐ Too expensive<br>☐ I don't see the need<br>☐ I treat them myself<br>☐ No veterinarians in my area<br>☐ Cultural beliefs<br>☐ Other
Specify	______________________________
707. Have you vaccinated your camel(s) in the past one year?	◯ Yes<br>◯ No
708. Against what diseases do you often vaccinate your camels?	_____________________________________
709. Did you pay for the vaccination?	◯ Yes<br>◯ No
710. How much do you pay on average for the vaccination?	______________________________<br>(Ksh.)
711. What prompts you to vaccinate your camels?	☐ Threat of a disease/disease outbreak<br>☐ Advice from veterinarian/health officers<br>☐ Required by law<br>☐ When vaccine is offered free<br>☐ I vaccinate when I can afford<br>☐ Other (Specify)
Specify	______________________________
712.Ectoparasites	
712a..Are ectoparasites a problem for your camels?	◯ Yes<br>◯ No
712b.If yes, which ectoparasites?	☐ Biting flies<br>☐ Ticks<br>☐ Mange<br>☐ Fleas
712c.If ticks, which control methods do you use?	☐ Pulll off ticks<br>☐ Use chemical<br>☐ Move calf paddocks<br>☐ Other(specify)

Specify	__________________
712d. If yes to use of Chemicals, which method?	☐ Dips<br>☐ Spot on<br>☐ Other(specify)
Specify	__________________
713. How often do you deworm your camels in a year?	◯ None<br>◯ Once<br>◯ Twice<br>◯ Thrice<br>◯ More than 3 times (Specify)
Please specify for more than 3 times deworming.	__________________
714.What are the three commonest camel health problems in your herd?	______________________
715. Has any of your camels died in the last one year?	◯ Yes<br>◯ No
If yes above, choose the age category and state the number below.	
Age category	☐ 0-6 months<br>☐ 7-12 months<br>☐ Above 1 year
Number of male dead between 0-6 months	__________________
Number of female dead between 0-6 months	__________________
Number dead male between 7-12 months	__________________
Number dead female between 7-12 months	__________________
Number dead male above 1 year	__________________
Number dead female above 1 year	__________________
716. What is the main cause of Death?	

0-6 months

- ◯ Got sick
- ◯ Natural disaster (e.g. drought)
- ◯ Predation
- ◯ Injury
- ◯ Don't know
- ◯ Other

Specify

____

What were the main clinical syndromes observed in the animals that got sick?

____

7-12 months

- ◯ Got sick
- ◯ Natural disaster (e.g. drought)
- ◯ Predation
- ◯ Injury
- ◯ Don't know
- ◯ Other

Specify

____

What were the main clinical signs observed in the animals that got sick?

____

13+ months

- ◯ Got sick
- ◯ Natural disaster (e.g. drought)
- ◯ Predation
- ◯ Injury
- ◯ Don't know
- ◯ Other

Specify

____

What were the main clinical signs observed in the animals that got sick?

____

717.Has any of your camels experienced the following in the last three months?

- ☐ Abortion
- ☐ Retained placenta
- ☐ Hygroma
- ☐ Swollen testicles
- ☐ Any other fertility problem
- ☐ None of the above

PART 8: HERDING STRATEGIES/PASTORALIST LIFE

801. What is the average distance covered in hours from the Boma to foraging area on a daily basis? (estimate)	◯ Less than 1 hour walk<br>◯ 1-2 hr walk)<br>◯ >2hr walk)
802. Who does the herding?	☐ Household Head<br>☐ Spouse<br>☐ Farm worker/Herder<br>☐ Son/Daughter<br>☐ Other (Specify)
Please specify the other persons doing herding.	__________________________________
803. Whose role is it to herd?	◯ Specific person<br>◯ Any person
804. Where does your camel(s) source water from?	☐ Private Well/watering point<br>☐ Communal well/watering point<br>☐ River/stream<br>☐ Other (Specify)
Please specify other source of water	__________________________________
805. Do you move with your camels from one place to another?	◯ Yes<br>◯ No
806. If yes above, how many times have you moved in the last one month?	◯ Once<br>◯ Twice<br>◯ Thrice<br>◯ More than 3 times (Specify below)
Moved more than 3 times	__________________________________
807. Why do you move with your camels?	☐ To look for pasture<br>☐ To look for water<br>☐ To escape disease/insect infestation<br>☐ Security reasons<br>☐ Its our way of life<br>☐ Other (Specify)
Specify other reason for moving	__________________________________
808. Who makes the decision to move the camels	◯ Herd owner/Self<br>◯ Elder(s)/Communal<br>◯ Other (Specify)
Specify other person/group who makes decision to move.	__________________________________
809. If NO, why? Please specify the reason(s)	____

Camel Sampling

Interviewer ID	◯ MC10 (Mutembei)<br>◯ MC11 (Denge)<br>◯ MC12 (Minayo)<br>◯ MC13 (Akoko)<br>◯ MC14 (Omollo)<br>◯ MC15 (Ngere)

Camel Details

1.0 Animal Name	__________________________________
2.0 Sex	◯ Male<br>◯ Female
3.0 Unit of Age	◯ Months<br>◯ Years
3.1Age (in Months)	__________________________________
3.2Age (in years)	__________________________________
4.0 Date of samplinf(dd/mm/yyyy)	__________________________________
5.0 Herd Type	◯ Mother/Home Herd<br>◯ Main Herd

Sample Collection

6.0 Please select all the specimens collected	☐ Milk<br>☐ Nasal swab<br>☐ Blood<br>☐ Vaginal swab
6.1 Milk Sample barcode	__________________________________
6.2 Nasal Swab specimen barcode	__________________________________
6.3 Blood specimen barcode	__________________________________
6.4 Vaginal swab specimen barcode	__________________________________
